# Comparative effectiveness of lifestyle interventions on cardiovascular risk factors among a Dutch overweight working population: A randomized controlled trial

**DOI:** 10.1186/1471-2458-11-49

**Published:** 2011-01-24

**Authors:** Johanna C Dekkers, Marieke F van Wier, Geertje AM Ariëns, Ingrid JM Hendriksen, Nico P Pronk, Tjabe Smid, Willem van Mechelen

**Affiliations:** 1 EMGO+ Institute and Department of Public and Occupational Health, VU University Medical Center, Van der Boechorststraat 7, 1081 BT Amsterdam, The Netherlands; 2 Body@Work, Research Center Physical Activity, Work and Health, TNO-VUmc, Van der Boechorststraat 7, 1081 BT Amsterdam, The Netherlands; 3 Municipal Health Service The Hague, PO Box 12652, 2500 DP, The Hague, The Netherlands; 4 TNO Quality of Life, P.O. Box 2215, 2301 CE Leiden, The Netherlands; 5 HealthPartners, JourneyWell, 8170 33rd Avenue South, Minneapolis, MN 55425, USA; 6 KLM Strategic Human Resources, AMS/SH, PO Box 7700, 1117 ZL Schiphol, The Netherlands

## Abstract

**Background:**

Overweight (Body Mass Index [BMI] ≥ 25 kg/m^2^) and obesity (BMI≥ 30 kg/m^2^) are associated with increased cardiovascular risk, posing a considerable burden to public health. The main aim of this study was to investigate lifestyle intervention effects on cardiovascular risk factors in healthy overweight employees.

**Methods:**

Participants were 276 healthy overweight employees (69.2% male; mean age 44.0 years [SD 9.2]; mean BMI 29.7 kg/m^2 ^[SD 3.1]). They were randomized to one of two intervention groups receiving a six month lifestyle intervention with behavior counseling by phone (phone group) or e-mail (Internet group), or to a control group receiving usual care. Body weight, height, waist circumference, sum of skinfolds, blood pressure, total cholesterol level and predicted aerobic fitness were measured at baseline, at 6 and at 24 months. Regression analyses included the 141 participants with complete data.

**Results:**

At 6 months a significant favorable effect on total cholesterol level (-0.2 mmol/l, 95%CI -0.5 to -0.0) was observed in the phone group and a trend for improved aerobic fitness (1.9 ml/kg/min, 95%CI -0.2 to 3.9) in the Internet group. At two years, favorable trends for body weight (-2.1 kg, 95%CI -4.4 to 0.2) and aerobic fitness (2.3 ml/kg/min, 95%CI -0.2 to 4.8) were observed in the Internet group.

**Conclusions:**

The intervention effects were independent of the used communication mode. However short-term results were in favor of the phone group and long-term results in favor of the internet group. Thus, we found limited evidence for our lifestyle intervention to be effective in reducing cardiovascular risk in a group of apparently healthy overweight workers.

**Trial registration:**

ISRCTN04265725

## Background

The prevalence of overweight (i.e., Body Mass Index [BMI] ≥ 25 kg/m^2^) and obesity (BMI ≥ 30 kg/m^2^) is high and still continues to increase. Overweight and obesity have become a worldwide epidemic, posing a considerable threat to public health [1,2]. Overweight is associated with increased risk for cardiovascular diseases and other health problems that are mainly cardiovascular risk factors [1]. In addition, it is responsible for high health care costs [3]. Therefore, early prevention of overweight and obesity is warranted.

It is now globally recognized that lifestyle modification aimed at improving dietary habits and physical activity is the first-line approach to reducing overweight and related cardiovascular risk [4-6]. The effect of lifestyle modification on cardiovascular risk is assumed to depend largely on weight loss [5].

Three recent reviews have shown favorable effects of lifestyle modification on body weight and/or cardiovascular risk factors in overweight or obese subjects [4,7,8]. Most research concerning the effects of lifestyle intervention on cardiovascular risk factors included subjects suffering from one or more overweight-related health problem(s). As yet, relatively few lifestyle intervention studies on the effect on cardiovascular risk factors have been performed in overweight/obese adults that were not selected for known co-morbidities [9-14]. Of these studies, three included only (peri- to postmenopausal) women[10,11,14] and one only men [9]. We were interested in lifestyle intervention effects on cardiovascular risk factors in overweight adults not selected for known co-morbidities. In this article we refer to them as apparently healthy overweight adults.

Recently, both the telephone[15-18] and the Internet/e-mail[19-22] have shown to be promising tools to deliver lifestyle interventions designed to enhance physical activity and/or nutrition behavior. Only a few of these studies investigated, apart from the effects on lifestyle, effects on cardiovascular risk factors in overweight subjects [17,21,22]. Furthermore, none of the distance-counseling studies used both the telephone and the Internet as an intervention mode, which would have allowed for a direct comparison between these two communication methods

As adults spend much of their time in the workplace, the worksite is regarded a suitable setting to promote healthy lifestyle changes to a large proportion of the population [23]. Moreover, the unfavorable changes in the worksite environment during the past decades (i.e., increase in vending machines and desk jobs), may have significantly contributed to unhealthier diet and sedentary behavior of employees [24]. We performed our study in an occupational setting. So far, only one Dutch high quality study regarding the effect of a lifestyle intervention program on health risk factors has been conducted among apparently healthy employees. However, the employees in that study were not overweight and the follow-up period was relatively short (9 months) [25].

To our knowledge, this is the first Dutch randomized controlled trial (RCT) to study the short- and long-term intervention effects of a distance-counseling lifestyle intervention program by phone and Internet/e-mail on cardiovascular risk factors in apparently healthy overweight workers. The main objective of this study was to evaluate the lifestyle intervention effects on cardiovascular risk factors at 6 and at 24 months. A second aim was to study whether these effects differed between the phone and Internet intervention modes and if adherence to the interventions was of influence on these effects.

## Methods

### Participants

The 276 participants in this study were a random sub-sample of 1386 apparently healthy overweight (BMI ≥ 25 kg/m^2^) subjects participating in a large-scale lifestyle intervention study, called ALIFE@Work [26]. In this randomized controlled trial (RCT) the effectiveness of a distance-counseling lifestyle intervention program, delivered by either phone or Internet/e-mail, was investigated in overweight employees.

Participants were employees from seven different companies (two IT-companies, two hospitals, an insurance company, the head office of a bank and a police force) located in The Netherlands. Inclusion criteria were: 1) ≥ 18 yrs old, 2) BMI ≥ 25 kg/m^2^, 3) access to Internet (at home or at work) and knowledge how to use it, 4) paid employment for at least 8 hours a week; 5) being able to read and write Dutch. Subjects who were pregnant, or were diagnosed or treated for disorders that made physical activity difficult were excluded.

The Medical Ethics Committee of the VU University medical center reviewed and approved the study design, procedure and informed consent procedure (December 11, 2003). All participants provided written informed consent. All subjects participated voluntarily and were free to cancel their participation at any time throughout the course of the study.

### Design and study procedures

A detailed description of the study procedures has been given elsewhere [26]. Briefly, the study procedure was as follows: all apparently eligible subjects received further study information and were invited to take part. Those who affirmed the invitation were invited to have their body weight and height measured near or at their worksite, in order to assess their BMI. Employees with a BMI < 25 kg/m^2 ^were subsequently excluded.

After baseline measurements (body weight and height), the 1386 employees subjects with a BMI ≥ 25 kg/m^2 ^were randomized to one of the three study groups and either to a group receiving basic weight measurements (80% of each study group) or to a group receiving additional measurements (i.e., waist circumference, sum of four skinfolds, blood pressure, total cholesterol level, and aerobic fitness) (20% of each study group). This two-step randomization meant that there were six groups an employee could be assigned to. Randomization to these six groups was done by block randomization, with each block containing 18 allocations. A computerized random number generator drew up an allocation schedule. An administrative assistant put the group allocation in opaque sealed envelopes, numbered 1 to 1,500. These envelopes were taken to the locations of the baseline measurements and opened in the given order. The researchers were blinded for the allocation schedule, but were not blinded for allocation after randomization. The participants were, in consequence of the nature of the intervention, not blinded for allocation after randomization. Employees were not allowed to change groups after randomization.

Follow-up measurements were done six months and two years after baseline. In addition to the measurements, participants completed surveys regarding their, among others, physical activity level, dietary habits, education, smoking status and medication use at all three time points. The surveys were sent to the home address of the participant approximately two weeks prior to the measurements. Data were collected from February 2004 till November 2006 at or near the participant's worksite.

### Interventions

All groups received self-help materials on overweight, physical activity and healthy diet by means of standard brochures issued by the Netherlands Heart Foundation, intended for the general public. Additionally, the phone and Internet group received a distance-counseling lifestyle intervention program. This intervention program was an adapted version of previous work of HealthPartners (Minnesota, USA) that was designed according to principles of cognitive-behavioral therapy [27]. The adaptation had involved translation to Dutch and to a Dutch tone of voice, and adaptations of cultural elements such as food and calorie charts, cooking methods, options when eating out and opportunities for everyday physical activity. The Dutch intervention was called 'Leef je Fit' (in English: 'Live Yourself Fit').

Leef je Fit, based on cognitive behavioral approach, consisted of ten educational modules that addressed physical activity and nutrition and taught lifestyle modification strategies (e.g., self-monitoring and goal-setting). Physical activities that could easily be fitted in daily life were encouraged (e.g., lunch-walking, active commuting), as well as a healthy diet with less fat, sugar and alcohol, and sufficient intake of fruit and vegetables. On the whole, the program emphasized sustainable lifestyle changes rather than weight loss. In each module, subjects were asked to complete several assignments related to the specific educational and behavioral foci of that module. The design of the program was such that subjects were able to finish any module within two weeks. The program was self-paced, but subjects had to finish the entire program within six months. All intervention subjects received personal tailored counseling support while working through the program. Counselors contacted participants in the phone group by phone to go through a module and to discuss the assignments. At the end of each call, an appointment for the next call in about two weeks was scheduled. When participants in the Internet group had completed a module their counselor received an automated e-mail about this. Thereafter the counselor checked the information the participant had provided and responded by e-mail within five working days. By way of automated e-mail reminders and, if the participant had selected this option, automated mobile phone text-messages, internet participants were encouraged to start and finish modules within two weeks. Thus, all participants had a maximum of ten counseling contacts during the intervention program. Counseling was done by four trained counselors (2 dieticians, two movement scientists) and according to two comparable standardized counseling protocols, one for each communication method.[26] Two weeks after randomisation, the counselor initiated the intervention by contacting the employees. Participants could also contact the counselor centre themselves.

### Outcome measures

All cardiovascular risk factors and body weight and height were measured according to protocols by trained research personnel [26]. *Waist circumference *(in cm) was measured twice with a tape measure (Gulick; Creative Health Products, Ann Arbor, MI, USA; range 0-150 cm) at the midpoint between the lower border of the ribs and the upper border of the iliac crest. Next, the two measurements were averaged. *Skinfold thicknesses *(in mm; subscapular, suprailiac, triceps and biceps) were measured twice on the right side of the body with a Harpenden caliper (HSK-BI; Baty International, Burgess Hill, UK; range 0-50 mm, graduation 0.2 mm). In case two measurements differed more than 1.0 mm, the skinfold was measured a third time. The value of the two (or three) obtained values was averaged. Next, the sum of the skinfolds at the four loci was computed. *Blood pressure *(in mmHg) was measured twice with a fully automated blood pressure monitor (Omron HEM 757 E [M5-I]; Omron Healthcare Europe BV, Hoofddorp, The Netherlands) after the participant had rested for 5 minutes in sitting position. This blood pressure monitor is validated and recommended for clinical use [28]. Approximately two minutes separated the two measurements during which the participant remained seated comfortably. Next, the mean value of the two measurements was computed. In case elevated (>140/90 mmHg) blood pressure levels were found, subjects were advised to visit their general practitioner.

*Total cholesterol level (TC) *was assessed in non-fasting capillary blood collected by finger stick. Blood was analyzed using a Reflotron^® ^Plus (Roche Diagnostics GmbH, Mannheim, Germany), which provides a good risk classification [29]. When a low (≤3.0 mmol/L) or elevated (≥ 6.5 mmol/L) TC level was found, a second assessment was completed and the two measurements averaged. Subjects with low or elevated TC level were advised to visit their general practitioner.

*Aerobic fitness level *was assessed by means of the submaximal Chester Step Test (CST) that has been shown to be a valid and reliable predictor of VO_2_max [30]. During the CST subjects were required to step on and off an adjustable gym bench. The height of the gym bench depended on the participant's age and current fitness level [31]. The test started at a relatively slow pace of 15 steps per minute. The pace increased every two minutes to respectively 20, 25, 30 and 35 steps per minute. A metronome was used to set the stepping rate. The test-instructor gave instructions throughout the test when necessary.

Subject's heart rate was monitored continuously with a heart rate monitor (Polar S610; Polar Electro Oy, Kempele, Finland). Also, the subject was asked to report his subjective rate of exertion at each increase in pace using a Borg scale [32]. The test was terminated at the end of a stage at which the subject's heart rate had reached 80% of his predicted maximal heart rate (220 minus age), or when the reported rate of perceived exertion exceeded 14 (hard) [31]. The estimated VO_2_max was calculated with software that came with the Chester step test (ASSIST creative resources Limited, Redwither Business Park, UK). The step test was chosen because of low cost, portability and ease of operation.

*Body weight *(kg) was measured using a digital scale (Seca 770; Seca GmbH & Co, Hamburg, Germany) with participants wearing light clothing and no shoes. *Body height *(cm) was measured with a portable stadiometer (Seca 214, Leicester Height Measure; Seca GmbH & Co, Hamburg, Germany). Weight and height were measured twice, and the mean value of the two measurements was computed. Next, the *Body Mass Index *(BMI) was calculated by dividing body weight (kg) by height squared (m^2^).

### Power calculation

A priori power calculations were done for DBP and for total cholesterol. The standard deviations (SD) were based on unpublished data from the Amsterdam Growth and Health Longitudinal study. The calculation to detect a change in DBP of 4.5 mmHg (SD 10.6 mmHg) with 80% power in two-tailed tests at a significance level of 0.05, determined the sample size for each study group at 87. The calculation to detect a change in total cholesterol of 0.4 mmol/l (SD 0.9 mmol/l) with 80% power in two-tailed tests at a significance level of 0.05, determined the sample size for each study group at 80. The sample size for this study was therefore determined at 300. Loss to follow-up was not taken into account.

### Statistical analyses

Linear regression analysis was used to evaluate the intervention effects on the cardiovascular risk factors. The cardiovascular risk factor level at six months or at 24 months was taken into the model as dependent variable and study group (phone, Internet and control) and baseline level of the risk factor as independent variables. Two dummy variables were created and coded such that the phone and Internet groups were compared with the control group. Subsequently, the phone and internet groups were compared: if the confidence interval of the phone group included the regression coefficient of the internet group and/or vice versa, the difference between the groups was not significant. To test whether adherence to the program influenced the intervention effects, number of counseled modules X study group was added to the model as interaction term.

All analyses were performed using SPSS software (version 12.0.1). P-values <0.05 were considered to be significant.

## Results

### Subjects

**Figure 1:**
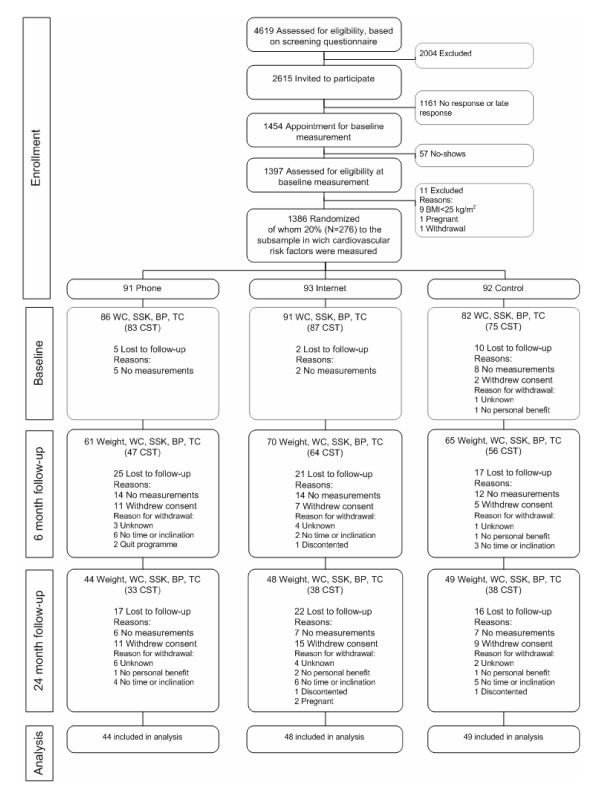
**Flow of subjects through the trial**. BP, blood pressure; CST, Chester Step Test; SSK, sum of skinfolds; TC, total cholesterol; WC, waist circumference.

Figure 1 shows the participant flow through the trial. It can be observed that 276 subjects (phone group: n = 91; Internet group: n = 93; control group: n = 92) of the 1386 eligible subjects were randomly assigned to the group in which cardiovascular risk factors were measured. Participants were predominantly male (69.2%), highly educated (56.9%) and non-smoking (87.2%) (Table 1). They had a mean BMI of 29.7 (SD 3.1) kg/m^2 ^and 36% was considered obese (i.e. BMI ≥ 30). Forty subjects (14.5%) were on medication for certain co-morbidities (hypertension (N = 26), hypercholesterolaemia (N = 13), diabetes mellitus (N = 10), depression (N = 7), history of cardiovascular events (heart infarction [N = 3]; angina pectoris [N = 1])). As shown in Table 1, no significant differences between the baseline characteristics existed between the three study groups.

**Table 1:** Baseline characteristics according to study group.

	Control (n = 92)	Phone (n = 91)	Internet (n = 93)	All (n = 276)	p-value
Male (%)	70.7	68.1	68.8	69.2	0.930

Age (SD), yrs	43.8 (9.4)	43.2 (9.6)	44.9 (8.5)	44.0 (9.2)	0.410

Body weight (SD), kg	92.3 ( 11.3)	94.4 (15.6)	94.0 (13.7)	93.5 (13.6)	0.540

Height (SD), cm	177.8 (9.0)	177.6 (10.1)	176.7 (8.4)	177.4 (9.2)	0.664

BMI (SD), kg/m^2^	29.2 (2.7)	29.8 (3.3)	30.0 (3.4)	29.7 (3.1)	0.141

Highly educated (%)	59.8	53.8	57.0	56.9	0.720

Smoking (%≥ 1 unit/day)	16.3	13.2	11.8	13.8	0.664

Medication for comorbidity (%)	15.2	15.4	12.9	14.5	0.907

Cardiovascular risk factors	n = 82	n = 86	n = 91	n = 259	

Waist (SD), cm	101.4 (8.9)	102.1 (10.9)	102.6 (9.7)	102.0 (9.9)	0.696

SSK (SD), mm	80.9 (24.1)	88.6 (28.8)	90.5 (29.8)	86.8 (28.0)	0.062

SBP (SD), mmHg	135.9 (15.0)	135.1 (15.3)	135.9 (16.6)	135.6 (15.6)	0.940

DBP (SD), mmHg	87.9 (10.8)	88.4 (9.9)	90.0 (10.0)	88.8 (10.2)	0.345

TC (SD), mmol/l^a^	5.0 (0.8)	4.9 (0.9)	4.9 (0.9)	4.9 (0.8)	0.769

Predicted VO_2_max (SD), ml/kg/min)^b^	38.9 (5.6)	37.6 (6.9)	36.7 (6.3)	37.7 (6.4)	0.092

Abbreviations: SD, standard deviation; BMI, body mass index; Waist, waist circumference; SSK, sum of skinfolds; SBP, systolic blood pressure; DBP, diastolic blood pressure; TC, total cholesterol; VO_2_max, maximal oxygen uptake, ^a ^n = 253 (control: n = 79, phone: n = 85, Internet: n = 89), ^b ^n = 245 (control: n = 75, phone: n = 83, Internet: n = 87).

Between baseline and two year follow-up, 47 participants were lost in the phone group, 45 participants in the Internet group, and 43 participants in the control group. Reasons for the loss to follow-up were (mainly) the lack of measurements and withdrawal of consent (Figure 1). The 141 subjects included in the study did not significantly differ from the 135 subjects that were lost to follow-up, except from being older and being more frequently highly educated (Table 2).

**Table 2:** Baseline differences between participants included^a ^in and excluded^b ^from the analyses.

	Included (n = 141)	Excluded (n = 135)	Difference (95% CI)	p-value
Male (%)	70.2	68.1	-	0.794

Age (SD), yrs	45.2 (9.1)	42.6 (9.1)	-2.6 (-4.8 to 0.4)	0.018

Body weight (SD), kg	92.2 (13.1)	94.9 (14.0)	2.8 (-0.4 to 6.0)	0.091

Height (SD), cm	176.9 (8.4)	177.8 (9.9)	0.9 (-1.3 to 3.1)	0.411

BMI (SD), kg/m^2^	29.4 (3.1)	30.0 (3.1)	0.6 (-0.2 to 1.3)	0.091

Highly educated (%)	63.1	50.4	-	0.039

Smoking (%≥ 1 unit/day)	12.1	15.6	-	0.485

Medication for comorbidity (%)^c^	13.5	15.6	-	0.607

Cardiovascular risk factors	n = 141	n = 118		

Waist (SD), cm	101.5 (10.0)	102.7 (9.8)	1.1 (-1.3 to 3.6)	0.357

SSK (SD), mm	84.8 (29.7)	89.3 (25.7)	4.5 (-2.4 to 1.4)	0.198

SBP (SD), mmHg	137.1 (16.0)	133.8 (15.1)	-3.3 (-7.2 to 0.5)	0.087

DBP (SD), mmHg	89.5 (10.6)	88.0 (9.7)	-1.4 (-3.9 to 1.1)	0.259

TC (SD), mmol/l^d^	5.0 (0.9)	4.9 (0.8)	-0.1 (-0.3 to 0.1)	0.334

Predicted VO_2_max (SD), ml/kg/min)^e^	37.4 (6.1)	38.1 (6.6)	0.7 (-0.9 to 2.3)	0.395

Abbreviations: SD, standard deviation; BMI, body mass index; Waist, waist circumference; SSK, sum of skinfolds; SBP, systolic blood pressure; DBP, diastolic blood pressure; TC, total cholesterol; VO_2_max, maximal oxygen uptake, ^a ^participants with complete measurements, ^b ^participants lost to follow-up due to the lack of measurements or withdrawal from the study, ^c ^n = 138 vs. n = 126, ^d ^n = 140 vs. n = 113, ^e ^n = 137 vs. n = 108.

### Adherence to intervention

**Figure 2:**
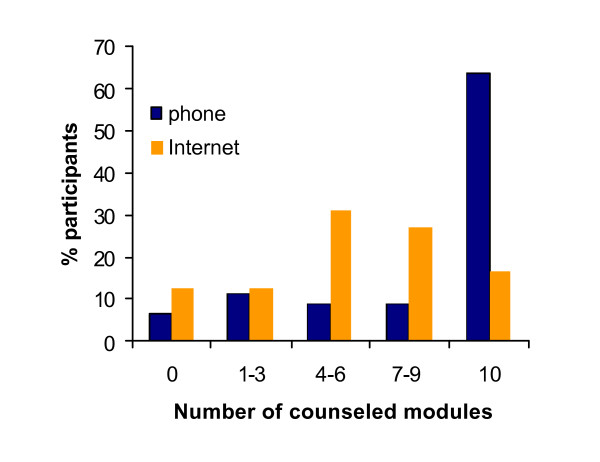
**Participation in the intervention**. The columns represent the proportion of participants in the phone and Internet groups that received no counseling (0) or that were counseled on 1-3, 4-6, 7-9 or 10 modules.

Figure 2 shows participation in the intervention for the study groups. In the phone group 6.8% never started the intervention (0 counseled modules) compared to 12.5% in the internet group. The proportion of subjects that was counseled on all modules was 64% in the phone group and 17% in the internet group. The median number of counseled modules was 4 (IQR = 2 to 4) in the phone group and 2 (IQR = 1.25 to 3) in the internet group. Analyses showed no significant interaction effects between the number of counseled modules and study group, indicating that the effect of the number of counseled modules was independent of the intervention group.

At 6 months, the number of counseled modules significantly affected body weight (-0.7 kg, 95%CI -0.9 to -0.4), waist (-0.6 cm; 95%CI -0.9 to -0.3), SBP (-0.8 mmHg; 95%CI -1.4 to -0.1), DBP (-0.6 mmHg; 95%CI -1.0 to -0.2) and SSK (-1.0 mm; 95%CI -1.9 to -0.1). At 24 months, no such significant effects were observed.

### Intervention effect on body weight and cardiovascular risk factors at 6 and 24 months

Table 3 shows the baseline, 6-months and two-year follow-up outcomes and differences between the study groups. Both at 6 and 24 months (Table 3), the intervention effects were not significant, except for total cholesterol in the phone group. This significant effect (-0.2 mmol/l, 95% CI -0.5 to -0.0) indicates that at 6 months TC level in the phone group was 0.2 mmol/l lower than in the control group. In addition, in the Internet group favorable trends were observed for aerobic fitness at 6 and 24 months (6 months: 1.9 ml/kg/min, 95%CI -0.2 to 3.9; 24 months: 2.3 ml/kg/min, 95%CI -0.2 to 4.8) and for body weight (-2.1 kg, 95%CI -4.4 to 0.2) at 24 months. Evaluation of the internet group against the phone group showed no significant differences.

**Table 3:** Baseline, 6-months and two-year follow-up outcomes and differences between the study groups.

	Controls (n = 49)			Phone (n = 44)			Internet (n = 48)			Phone T6	Phone T24	Internet T6	Internet T24
	**Baseline**	**T6**	**T24**	**Baseline**	**T6**	**T24**	**Baseline**	**T6**	**T24**	**Change vs. control** **(95% CI)**	**Change vs. control** **(95% CI)**	**Change vs. control** **(95% CI)**	**Change vs. control** **(95% CI)**

Body weight (SD), kg	91.5 (10.6)	89.8 (10.4)	90.3 (10.8)	91.7 (13.6)	89.3 (13.7)	90.3 (14.8)	93.3 (15.0)	90.5 (15.3)	90.0 (15.4)	-0.7 (-2.7 to 1.2)	-0.3 (-2.6 to 2.0)	-1.0 (-2.9 to 0.9)	-2.1 (-4.4 to 0.2)

Waist (SD), cm	101.7 (8.5)	99.2 (9.3)	99.3 (9.7)	99.9 (10.2)	96.4 (11.0)	97.9 (11.0)	102.9 (11.1)	99.4 (11.4)	99.3 (11.9)	-1.1 (-3.1 to 0.9)	0.3 (-2.0 to 2.6)	-1.0 (-2.9 to 0.9)	-1.1 (-3.3 to 1.1)

SSK (SD), mm ^a^	77.8 (24.8)	72.5 (25.5)	75.6 (19.1)	82.3 (24.5)	73.5 (30.4)	81.0 (25.7)	94.1 (36.0)	89.2 (41.8)	83.7 (28.7)	-2.4 (-8.4 to 3.7)	0.1 (-5.3 to 8.1)	0.1 (-6.0 to 6.2)	-3.3 (-10.1 to 3.5)

SBP (SD), mmHg	135.2 (11.4)	134.2 (14.2)	133.7 (15.3)	138.2 (15.6)	136.4 (17.0)	136.4 (15.9)	138.2 (19.9)	137.7 (15.2)	135.4 (16.5)	0.1 (-4.3 to 4.6)	0.7 (-4.2 to 5.7)	1.5 (-2.9 to 5.8)	-3.0 (-5.1 to 4.5)

DBP (SD), mmHg	87.0 (9.5)	85.5 (9.9)	84.6 (9.8)	89.6 (10.4)	88.4 (11.2)	86.9 (12.0)	91.8 (11.4)	90.2 (9.8)	88.1 (10.5)	1.0 (-2.0 to 4.0)	0.5 (-2.8 to 3.8)	1.3 (-1.7 to 4.2)	0.2 (-3.1 to 3.5)

TC (SD), mmol/l	4.9 (0.8)	5.1 (0.6)	4.9 (0.8)	4.9 (0.9)	4.9 (0.9)	4.8 (1.0)	5.1 (0.9)	5.2 (0.9)	5.0 (1.0)	-0.2 (-0.5 to -0.0)	-0.0 (-0.3 to 0.3)	-0.1 (-0.3 to 0.1)	-0.1 (-0.4 to 0.2)

VO_2_max (SD), ml/kg/min	n = 49 38.8 (5.7)	n = 45 39.2 (4.8)	n = 39 39.7 (7.6)	n = 44 37.6 (6.6)	n = 37 38.7 (7.8)	n = 37 38.8 (8.5)	n = 48 36.3 (5.0)	n = 45 39.1 (6.4)	n = 40 39.6 (7.2)	0.5 (-1.6 to 2.6)	0.2 (-2.3 to 2.8)	1.9 (-0.2 to 3.9)	2.3 (-0.2 to 4.8)

Abbreviations: SD, standard deviation; Waist, waist circumference; SSK, sum of skinfolds; SBP, systolic blood pressure; DBP, diastolic blood pressure; TC, total cholesterol; VO_2_max, maximal oxygen uptake. ^a ^For seven subjects (control: n = 2, phone: n = 2, Internet: n = 3) one or more skinfolds were outliers, apparently due to measurement errors. These subjects were excluded from the analyses.

## Discussion

The main aim of this study was to evaluate lifestyle intervention effects on cardiovascular risk factors at 6 and at 24 months in a Dutch overweight working population. Two features of our RCT were that 1) we included apparently healthy overweight workers and that 2) we examined simultaneously the efficacy of two intervention modes, i.e., phone and Internet, to deliver the intervention.

Our results indicate limited effectiveness of the lifestyle intervention in modifying cardiovascular risk in overweight subjects. This contrasts with significant lifestyle intervention effects on cardiovascular risk factors observed in overweight adults in other studies [4,8]. It has been reported that intervention effects on cardiovascular risk factors occur mainly in overweight subjects with cardiovascular risks [7]. In the majority of the studies the overweight subjects were at increased cardiovascular risk, whereas our overweight subjects were apparently healthy without increased cardiovascular risk. This may explain the absence of intervention effects in our study. Lifestyle intervention studies including apparently healthy overweight/obese subjects and overweight/obese subjects with one or more co-morbidities are needed to confirm this hypothesis.

Another explanation for the limited effectiveness may be that the lifestyle intervention was not intense enough to establish and maintain significant changes in the cardiovascular risk factors. Our intervention program aimed at promoting a healthy lifestyle by stimulating subjects to meet the Public Health guideline of PA and to consume a healthy diet, i.e., at least two pieces of fruit and 150-200 grams of vegetables per day. Vegetable and fruit intake in our subjects was already close to the public health guidelines [33], and thus no lifestyle change could be expected due to a ceiling effect.

Also, the ten counseling sessions on a two-weekly basis may have formed an inadequate level of guidance compared to the number of counseling sessions in other studies [22,34]. Furthermore, no additional significant intervention was provided following the initial active treatment phase during the first 6 months. The absence of continued contact over the remaining 18 months may have eroded initially adopted changes and reduced the likelihood for retention of short-term effects. More information is needed on the optimal number of contacts necessary to enhance and maintain lifestyle modification.

Although most results at 6 months were not significant or clinically meaningful, most effects on cardiovascular risk factors were in the expected direction and, except for aerobic fitness, in favor of the phone group. Interestingly, at 24 months the intervention effects were in favor of the internet group. This may be explained by the fact that from 6 to 24 months the internet group lost another 1.1 kg of weight (-2.1 minus -1.0) compared to the control group, whereas the phone group gained 0.5 kg of weight (-0.3 minus -0.7) in the same period. This finding provides support for the assumption that the effect of lifestyle modification on cardiovascular depends on weight loss [5]. The lack of significant intervention effects may be due to the limited statistical power of our study, due to the high loss to follow-up.

A second aim of our study was to evaluate whether the intervention effects on cardiovascular risk factors were dependent on the communication mode used to deliver the program. As so far, no direct comparison between phone- and e-mail counseling had taken place, evidence to support a hypothesis about the superiority of either mode of counseling was not available.

We found no evidence for one of the two communication modes to be more effective than the other. However, effects at 6 months were in favor of counseling by phone and at 24 months in favor of e-mail counseling. As e-mail counseling lacks contact with a person, it may have been perceived as being more impersonal or having a lack of social support [35]. Also, e-mail contacts contain no emotional cues, which make it less easy to establish a bond than phone counseling. Consequently, e-mail counseling may have been less effective for supporting behavior change than counseling by phone, resulting in more favorable intervention effects in the phone group at 6 months. However, counseling stopped after these six months. Due to the cessation of personal contact the phone group may have experienced more difficulties in continuing to adopt a healthy lifestyle than the internet group, resulting in more favorable results in the internet group at 24 months. Also, participants in the internet group were able to read their email conversations again and again, whereas verbal conversations in the phone group could have been easily forgotten. Further lifestyle intervention studies that involve both phone and internet to deliver the lifestyle intervention are needed to increase the understanding of these communication modes to deliver interventions.

We also found that independent of the communication mode, the more modules completed, the stronger the intervention effects on the cardiovascular risk factors. Adherence has been reported to be positively associated with weight loss [16]. Thus, it is conceivable that a higher adherence in our subjects would have resulted in greater weight loss and, consequently, in stronger intervention effects. Effort should be taken to get insight into ways to increase adherence in long-term lifestyle studies aimed at reducing weight and cardiovascular risk. Future studies should improve the adherence of participants to the trial.

Several limitations in this study need consideration. First, as has been found in other lifestyle modification long-term studies [7], we had a high proportion (135/276) of subjects lost to follow-up, which may have considerably affected our results. However, as reported earlier, the 135 subjects lost to follow-up did not significantly differ from the 141 subjects in the study regarding demographics, anthropometrics and cardiovascular risk factors, except from being younger and less often highly educated. Despite this, intervention effects may have been overestimated because the 141 subjects we based our results on may have experienced greater weight loss than subjects lost to follow-up. Therefore the results should be interpreted with caution. The main reason why participants were lost to follow-up in our study was the missing of one ore more measurements (see Flow diagram), which may have been partly due to the fact that it appeared hard to schedule an appointment for the extra measurements in between liabilities at work. The other reason for the (high) loss to follow-up was withdrawal from the study. Several reasons for withdrawal were reported, among which no personal benefit and lack of time. Restricting the outcomes of interest to decrease the burden for participants and stressing the need of commitment to complete the trial could help to reduce the high loss to follow-up.

A second limitation is that we limited our analyses to participants with complete data. Additional analyses (results not shown) indicated that, in the intervention groups, participants that completed follow-up measurements had also completed more modules compared to participants that were excluded from the analyses due to missing follow-up measurements. As argued before, this may have resulted in greater weight loss and consequently in more favorable changes in CV risk factors in participants included in the study than in those excluded from the analyses. Again, caution should be taken when interpreting the results.

Third, the voluntary participation in this study may have resulted in selection bias. It is conceivable that the overweight subjects taking part in our study were more willing to change their lifestyles, were more interested in PA and healthy eating and were more conscious about their health than overweight subjects that did not participate in the study. Despite this possible selection bias, the voluntary participation may have contributed to high external validity.

Fourth, our subjects are not representative of the Dutch working population of which 57% is male and 40% highly educated [36]. This is due to the fact that the majority of companies that participated in this study employ predominantly men and white collar workers. Consequently, the generalizability of the study is limited.

Fifth, some of our subjects used medication for hypertension and hypercholesterolaemia, which may have affected the intervention effects on blood pressure and cholesterol level. Due to the relatively small number of subjects using medication for the different co-morbidities, we could not reliably check whether medication use has influenced the intervention effects.

These limitations should be balanced against several strengths of the study. First, subjects were recruited from a variety of companies making the population studied a more heterogeneous group than solely relying on recruitment from a single employer group. Next, the random selection of a subset of subjects in each of the study groups allowed for an in-depth investigation of the intervention effects on measured, as opposed to self-reported, cardiovascular outcomes. This novel approach to risk factor modification in a sample of apparently healthy workers allowed for robust conclusions to be drawn from this investigation.

## Conclusions

In conclusion, the results of this study provide limited evidence for lifestyle interventions being effective in establishing favorable short- and long-term changes in cardiovascular risk factors in a group of apparently healthy overweight workers. Although the majority of effects were not significant or clinically meaningful, they were all in the expected direction and are therefore likely of significance and interest to public health. The intervention effects were independent of the communication mode deployed, although short-term results were in favor of the phone group and long-term results in favor of the Internet group. Additional research, especially long-term trials, involving apparently healthy overweight subjects as well as different communication modes to deliver lifestyle modification, are needed to improve our understanding of lifestyle intervention effects on cardiovascular risk factors in apparently healthy overweight subjects.

## Competing interests

The authors declare that they have no competing interests.

## Authors' contributions

JCD participated in conception and design of the study, acquisition of data, analysis and interpretation of data and drafted the manuscript. MFW participated in the conception and design of the study, the acquisition of data and critically revised the manuscript. GAMA is co-applicant of the grant and provided advice and guidance on the study design and the conduct of the study. IJMH and NPP advised on the study design and critically revised the manuscript. TS helped to recruit companies, advised on the study design and critically revised the manuscript. WM obtained funding, supervised the study, and critically revised the manuscript. All authors read and approved the final manuscript.

## Pre-publication history

The pre-publication history for this paper can be accessed here:

http://www.biomedcentral.com/1471-2458/11/49/prepub
